# A switch in nucleotide affinity governs activation of the Src and Tec family kinases

**DOI:** 10.1038/s41598-017-17703-5

**Published:** 2017-12-12

**Authors:** Freia von Raußendorf, Anita de Ruiter, Thomas A. Leonard

**Affiliations:** 1 Department of Structural and Computational Biology, Max F. Perutz Laboratories (MFPL), Campus Vienna Biocenter 5, 1030 Vienna, Austria; 2 Institute of Molecular Modeling and Simulation, University of Natural Resources and Life Sciences (BOKU), 1190 Vienna, Austria; 3 Department of Medical Biochemistry, Medical University of Vienna, 1090 Vienna, Austria

## Abstract

The Tec kinases, closely related to Src family kinases, are essential for lymphocyte function in the adaptive immune system. Whilst the Src and Abl kinases are regulated by tail phosphorylation and N-terminal myristoylation respectively, the Tec kinases are notable for the absence of either regulatory element. We have found that the inactive conformations of the Tec kinase Itk and Src preferentially bind ADP over ATP, stabilising both proteins. We demonstrate that Itk adopts the same conformation as Src and that the autoinhibited conformation of Src is independent of its C-terminal tail. Allosteric activation of both Itk and Src depends critically on the disruption of a conserved hydrophobic stack that accompanies regulatory domain displacement. We show that a conformational switch permits the exchange of ADP for ATP, leading to efficient autophosphorylation and full activation. In summary, we propose a universal mechanism for the activation and autoinhibition of the Src and Tec kinases.

## Introduction

The Tec family kinases Btk and Itk are essential for B- and T-lymphocyte development in the adaptive immune system. Engagement of the B- or T-cell receptor triggers a cascade of intracellular phosphorylation events, initiated by phosphorylation of ITAM motifs in the cytoplasmic tails of activated receptors by Src family kinases^1–3^. The phosphorylated ITAMs recruit the tandem SH2 domain tyrosine kinases Syk and ZAP-70 in B- and T-cells respectively, which leads to activation of Tec family tyrosine kinases upon their PI3K-dependent recruitment^4^. Activated Btk and Itk phosphorylate phospholipase C-γ^5,6^, which triggers calcium flux in the cell by producing the second messenger inositol-1,4,5-trisphosphate (IP3). Dysregulation of Btk in humans causes the heritable disorder X-linked agammaglobulinemia^7^, characterised by a lack of circulating antibodies and a corresponding susceptibility to opportunistic infection. Itk dysfunction, on the other hand, specifically affects the development of T-cells and has been implicated in a number of lymphoproliferative disorders^8–10^, as well as playing a role in HIV replication and infectivity^11,12^.

Whilst the Tec and Src kinases are often referred to as separate families, they are in fact very closely related, belonging to the same branch of the tyrosine kinase phylogenetic tree^13^ and sharing in common a conserved SH3-SH2-kinase domain module. Src kinases are localised to the plasma membrane via lipid modification of their N-termini, whereas Tec kinases are regulated by the lipid second messenger PIP_3_, responding specifically via their N-terminal PH domain^14^. Src and Tec kinases are activated by engagement of intracellular ligands for their SH2 and SH3 domains, events that promote activation loop phosphorylation and downstream signalling^15–17^. Deletion of the PH domain or mutation of the phosphotyrosine-binding SH2 domain of Itk results in a loss of Itk activation^16,18^.

The structures of Src, Hck, and Abl kinases show a conserved intramolecular assembly of their regulatory SH3, SH2, and kinase domains that maintains the kinase in an autoinhibited conformation^19–21^. While Src and Hck are proposed to be maintained in this conformation by intramolecular engagement of their phosphorylated C-terminal tail^20,21^ and N-terminal myristoylation of Abl stabilises a similar conformation^19^, the Tec kinases notably lack either feature, leading to proposals that the PH domain might fulfil this function^22,23^. However, the recent crystal structure of the SH3-SH2-kinase domains of Btk shows a similar domain arrangement to Src in the absence of both the PH domain and a C-terminal tail^23^. The interface between the regulatory and kinase domains is mediated by a variable linker between the SH2 and kinase domains that forms a network of interactions essential for maintaining the inactive state. Displacement of the SH3 domain of Hck activates the kinase by relieving these inhibitory constraints^15,17^ and promoting a conformational switch in the N-terminal end of the catalytic domain^24^.

Activation loop autophosphorylation regulates the activity of Src tyrosine kinases^17,25,26^. In the Tec kinases, activation loop phosphorylation in cells depends on the activity of upstream Src kinases^27–30^, presumably via recruitment and activation of the kinase by SH2 and SH3 domain-ligands. Like the Src kinases, the Tec kinases robustly autophosphorylate *in vitro* ^16,23,31^. However, while the Src kinases have been extensively studied, the Tec kinases have been suggested to be mechanistically distinct, despite their evolutionary similarity.

We set out to determine the structure and mechanism of activation of the T-cell kinase Itk. To our surprise, we discovered that both Itk and Src bind ADP significantly stronger than the non-hydrolysable ATP analogue, AMPPNP. We found that activation loop phosphorylation or C-terminal tail deletion had no effect on the nucleotide binding preference or autoinhibited conformation of Src in solution. We describe the crystal structure of autoinhibited Src bound to ADP and use molecular dynamics to rationalise the binding preference for ADP. We show that Itk adopts a stable, globular conformation in the absence of its PH domain that is well described by the autoinhibited state of Src. We demonstrate the allosteric activation of both Itk and Src by mutation of a conserved hydrophobic stack between the regulatory and kinase domains, which converts the kinase from an ADP- to an ATP-binding conformation that undergoes robust auto-phosphorylation. In summary, we present a general mechanism for the allosteric activation and autoinhibition of both Src and Tec family tyrosine kinases.

## Results

### Autoinhibited Itk and Src kinases preferentially bind ADP

**Figure 1:**
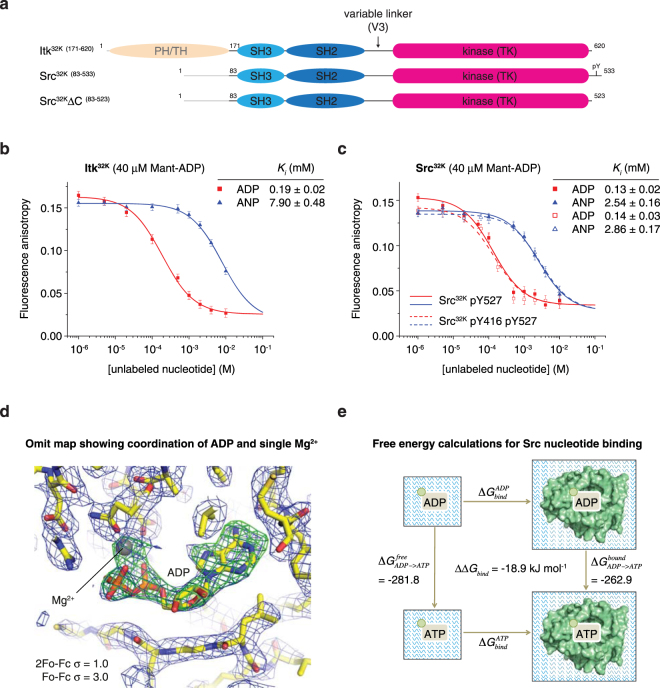
Autoinhibited Itk and Src kinases preferentially bind ADP. (**a**) Schematic of constructs evaluated in this study. Itk^32K^, Src^32K^, and Src^32K^ΔC contain the conserved module of SH3, SH2, and kinase (^32K^) domains. Src^32K^ was truncated by 10 amino acids to remove its C-terminal tail (inclusive of the Y527 phosphorylation site), generating a construct we refer to as Src^32K^ΔC (ΔC-tail). (**b**) Competition nucleotide displacement assay for Itk^32K^. 40 μM Itk^32K^ was incubated with 40 μM Mant-ADP until equilibrium was established. Unlabelled ADP or AMPPNP was titrated into the reaction and fluorescence anisotropy monitored. Comparison of the equilibrium inhibition constants (*K*_*i*_) gives an indication of the relative strength of nucleotide binding. AMPPNP binds to Itk^32K^ with 42-fold weaker affinity. (**c**) Competition nucleotide displacement assay for Src^32K^. 40 μM tail-phosphorylated Src^32K^ (pY527) or doubly phosphorylated Src^32K^ pY416 (pY527) was incubated with 40 μM Mant-ADP until equilibrium was established. Unlabelled ADP or AMPPNP was titrated into the reaction and fluorescence anisotropy monitored. Solid lines correspond to Src^32K^, dashed lines to Src^32K^ pY416. AMPPNP binds to Src^32K^ with 20-fold weaker affinity than ADP. Activation loop phosphorylation (dashed lines) does not affect the nucleotide-binding properties of Src^32K^. (**d**) Omit map showing clear electron density for ADP and a single magnesium ion in the nucleotide-binding pocket of Src. The magnesium is coordinated by Asn391, Asp404, and the α- and β- phosphates of ADP. The β-phosphate is directly coordinated by the guanidinium side chain of Arg388. (**e**) Thermodynamic cycle used for molecular dynamics calculations. The binding processes of ADP or ATP moving from bulk water to the bound state within Src kinase is computationally expensive to calculate. Instead we make use of a thermodynamic cycle. Because free energy is a state function, we can determine the relative binding free energy from the vertical arrows, where we convert ADP to ATP both when in solution and when bound to Src kinase.

Sequence and structural homology of Itk to Src (Fig. 1a) indicated that its biochemical properties, structure, and mechanism of activation were likely to also be similar. The first crystal structures of Src and Hck in their autoinhibited conformations suggested that nucleotide occupancy of the active site promoted ordering of the activation loop in its inactive conformation^20,21^. Subsequent structures of Src and Btk under different crystallisation conditions, however, have revealed an ordered conformation of the activation loop independent of nucleotide and Mg^2+^ binding^23,32^.

To characterise the nucleotide binding of Itk and Src, we purified recombinant wild type and mutant constructs of Itk and Src containing their respective SH3, SH2, and kinase domains (from here on referred to as Itk^32K^, Src^32K^, and Src^32K^ΔC, Fig. 1a). We confirmed the mass and homogeneity of isolated species by high-resolution anion exchange chromatography, intact mass spectrometry, and Western blotting with phospho-specific antibodies (Supplementary Figs 1–3). We performed fluorescence anisotropy measurements using Mant-labelled nucleotides. Structural modelling of Mant-ADP bound to Src did not reveal any steric clashes or additional contacts with the Mant moiety (Supplementary Fig. 4a,b). To avoid autophosphorylation during the course of the titration, we used the non-hydrolysable ATP analogue AMPPNP. Itk^32K^ binds ADP with a *K*_*d*_ of 23.7 ± 1.7 μM, while AMPPNP was estimated to bind with 15-fold lower affinity (Supplementary Fig. 4c) (in the absence of saturating data for AMPPNP, the upper asymptote of the curve was set to the same value as for ADP based on the maximum anisotropy achievable being the same in each case). Src^32K^ bound ADP approximately 6-fold more tightly than AMPPNP (Supplementary Fig. 4d).

Since the concentration of ATP in the cell is typically higher than ADP^33,34^, we performed nucleotide competition assays in order to determine which nucleotide is bound *in vivo*. We measured the concentrations of ADP or AMPPNP required to displace Mant-ADP. The equilibrium inhibition constant for displacement of ADP by AMPPNP in the case of Itk^32K^ is 7.90 mM, approximately a 41-fold excess over the concentration of ADP required (Fig. 1b). Src^32K^ exhibits a similar 19-fold preference for ADP over AMPPNP while activation loop phosphorylated Src^32K^ exhibits identical behaviour, indicating that activation loop phosphorylation alone does not change the binding preference of Src for ADP (Fig. 1c).

The finding that Itk and Src preferentially bind ADP is qualitatively supported by thermal stability measurements showing that ADP stabilises Itk^32K^ and Src^32K^ to a greater extent at the same nucleotide concentrations (Supplementary Fig. 4e,f), irrespective of activation loop phosphorylation (Supplementary Fig. 4 g). We noted that the thermal stability of Src^32K^ initially decreases at low concentrations of ATP, before rising again at higher concentrations. This was reproducible and correlates with autophosphorylation of Tyr416 during the course of the assay (Supplementary Fig. 4h).

To investigate the preferred binding of ADP at atomic resolution we determined the crystal structure of tail-phosphorylated Src in the presence of ADP and magnesium. Src^32K^ crystallised in the same conditions as previously reported for the nucleotide-free protein and in the same space group (Supplementary Fig. 5a)^21^. The overall structure agrees closely with that of a previous Src structure in complex with AMPPNP (2SRC), in which the activation loop is fully ordered^32^ (Supplementary Fig. 5b). A difference density map of the nucleotide-binding site shows clear electron density for the adenosine, both alpha and beta phosphates, and a single magnesium ion (Fig. 1d). The magnesium ion is coordinated between the alpha and beta phosphates and Asn391 and Asp404. The beta phosphate is directly coordinated by Arg388, the guanidinium group of which is stabilised by a hydrogen bond network between Asn391, Asp386 and Arg388 (Supplementary Fig. 5c,d). For comparison, the coordination of AMPPNP in the active conformation of insulin receptor tyrosine kinase (IRK) is shown in Supplementary Fig. 5e,f.

While the position of the alpha phosphate is superimposable with that of AMPPNP and the protein has, in principle, a large enough pocket in which to accommodate an additional phosphate, our binding experiments indicate a strong preference for ADP over AMPPNP (Fig. 1b,c). In order to rationalise the energetic basis for ADP over ATP binding, we computed the relative binding free energies between ADP and ATP using molecular dynamics simulations^35–37^. Figure 1e shows a schematic overview of the thermodynamic cycle used in this approach (see also Supplemental Text). The perturbation of ADP to ATP in the bound state resulted in a free energy difference of -262.9 kJ mol^−1^ compared with −281.8 kJ mol^−1^ in the free state (Fig. 1e). This results in a relative binding free energy for ADP over ATP of −18.9 kJ mol^−1^, confirming our experimental findings.

### Itk adopts a Src-like autoinhibited conformation

**Figure 2:**
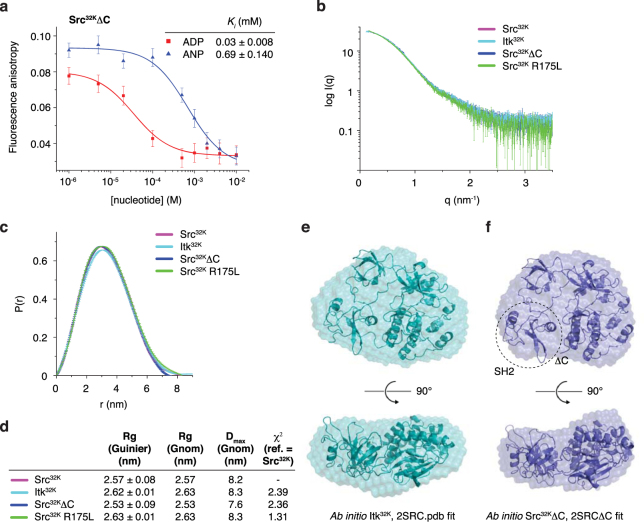
Itk adopts a Src-like autoinhibited conformation. (**a**) Competition nucleotide displacement assay for Src^32K^ΔC. 40 μM Src^32K^ΔC was incubated with 40 μM Mant-ADP until equilibrium was established. Unlabelled ADP or AMPPNP was titrated into the reaction and fluorescence anisotropy monitored. AMPPNP binds to Src^32K^ with 23-fold weaker affinity than ADP. (**b**) Small angle X-ray scattering curves for Itk^32K^ (cyan), Src^32K^ (magenta), Src^32K^ΔC (blue), and Src^32K^ R175L (green). (**c**) Pair distribution functions for Itk^32K^ (cyan), Src^32K^ (magenta), Src^32K^ΔC (blue), and Src^32K^ R175L (green) indicate that the particles exhibit almost identical radii of gyration and maximum dimensions. (**d**) Table of physical parameters obtained from the scattering curves for Itk^32K^, Src^32K^, Src^32K^ΔC, and Src^32K^ R175L. (**e**)*Ab initio* model of Itk^32K^ and docking of autoinhibited Src (2SRC.pdb) into the molecular envelope. Two orientations of the envelope are shown for clarity. (**f**) *Ab initio* molecular envelope for Src^32K^ΔC illustrates the fit of the crystal structure of autoinhibited Src.

C-terminal tail phosphorylation of the Src kinases Src, Hck, and Lck has previously been proposed to stabilise and maintain the inactive, autoinhibited conformation of the kinase^20,21,32,38^, while the PH domain of Btk has been proposed to stabilise its inactive conformation^22,23^. Having observed that Itk^32K^ binds and is stabilised by ADP in the absence of a C-terminal tail, we investigated whether the C-terminal tail of Src influences its nucleotide binding or conformation *in vitro*, using a construct of Src lacking its C-tail (Src^32K^ΔC) and a SH2 domain mutant of Src that is incapable of binding phosphotyrosine peptides (Src^32K^ R175L, Supplementary Fig. 3a,b)^39^. Src^32K^ΔC binds ADP with approximately 23-fold higher affinity than AMPPNP (Fig. 2a), indicating that tail dephosphorylation does not affect the preference of Src for ADP over ATP (Src^32K^ exhibits 19-fold stronger binding to ADP, Fig. 1c). Thermal stability measurements support the stabilisation of Src^32K^ΔC by ADP (Supplementary Fig. 6a).

To address whether Itk^32K^, Src^32K^, Src^32K^ΔC, and Src^32K^ R175L adopt the same intramolecular conformation, we collected small-angle X-ray scattering (SAXS) data on all four proteins in solution in the presence of ADP and magnesium (Fig. 2b). The scattering curves and pair distribution functions of Itk^32K^, Src^32K^, Src^32K^ΔC, and Src^32K^ R175L are superimposable (Fig. 2b,c). The structural parameters of the four particles are summarised in Fig. 2d and Supplementary Fig. 6b-e. Fitting of the theoretical scattering curve for autoinhibited Src (PDB entry: 2SRC)^32^ to the experimental scattering of Src^32K^ confirms that Src adopts an identical conformation in solution (χ^2^ = 0.22, Supplementary Fig. 6f). Iterative *ab initio* calculation of the molecular envelopes from the pair-distribution functions converged on a core electron density distribution in each case, which is well fitted by the crystal structure of Src. Superposition of the molecular envelopes (Fig. 2e,f, Supplementary Fig. 6f) indicates that these proteins adopt a very similar shape and conformation in solution and this is confirmed by the close agreement between the experimental scattering curves (Itk^32K^ χ^2^ = 2.39, Src^32K^ΔC χ^2^ = 2.36, Src^32K^ R175L χ^2^ = 1.31; 0.16 ≤ q ≤ 4 nm^−1^).

Having observed that activation loop phosphorylation did not influence the nucleotide binding of Src^32K^ (Fig. 1c), we next addressed whether it altered the overall conformation of Src^32K^. The experimental scattering curve and pair distribution function indicate that, at the resolution of the SAXS data (nominally 18 Å), this protein adopts the same conformation as Src^32K^ in solution (Supplementary Fig. 6g-i). Together, these findings indicate that Itk^32K^ adopts a Src-like autoinhibited conformation in solution and that the C-terminal tail is dispensable for assembly of the closed conformation of Src.

### An allosteric switch triggers nucleotide exchange

**Figure 3:**
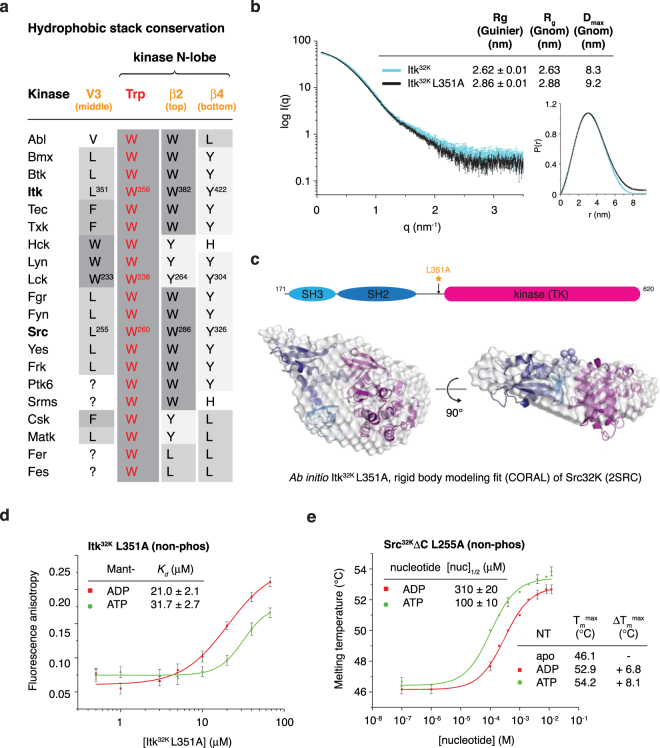
An allosteric switch triggers nucleotide exchange. (**a**) Hydrophobic stack conservation in Src family kinases. The table illustrates the amino acid identity at each of the three positions in the hydrophobic stack in addition to the invariant tryptophan (Trp260 in Src) for all Src family kinase members. The positions are presented according to their N- to C-terminal order in the primary sequences. Where it was not possible to unambiguously determine the identity of the amino acid contributed by the SH2-kinase variable linker (V3), the residue is denoted by a question mark. (**b**) Small angle X-ray scattering of Itk^32K^ L351A (black). The scattering curve for wild type Itk^32K^ (cyan) is overlaid for comparison. Calculation of the pair distribution function for Itk^32K^ L351A shows an increase in the radius of gyration from 2.63 nm to 2.88 nm and an increase in D_max_ from 8.3 to 9.2 nm compared to wild type Itk^32K^. (**c**) Rigid body modelling of Itk^32K^ L351A and fit to *ab initio* molecular envelope. Displacement of both the SH3 and SH2 domains is required for a good fit to the experimental scattering curve. (**d**) Determination of the binding constants of Itk^32K^ L351A for nucleotides by fluorescence anisotropy. Non-phosphorylated Itk^32K^ L351A binds ADP with similar affinity (21 μM) to wild type Itk^32K^ (20 μM) and binds ATP (32 μM) with slightly weaker, but comparable affinity, to ADP. (**e**) Determination of the thermal stabilities and relative binding affinities of non-phosphorylated Src^32K^ΔC L255A for nucleotides by differential scanning fluorimetry. Src^32K^ΔC L255A binds ATP with higher affinity (100 μM) than ADP (310 μM), while ATP also confers an additional 1.3 °C of thermal stability over ADP.

The inactive conformation of Src is stabilised by a conserved hydrophobic stack on the distal surface of the N-lobe of the kinase domain in which a hydrophobic side chain is contributed by the linker between the regulatory domains and the kinase domain^40,41^. Sequence analysis shows that this residue is Leu, Trp, or Phe in all Src, Abl, Tec, Csk, and Fer family kinases, which derive from the same branch of the human kinome^13^ (Fig. 3a, Supplementary Fig. 7a). The inactive conformation is stabilised by the insertion of the side chain of the invariant tryptophan (W260) into a hydrophobic pocket formed between the αC helix and strand β4 of the kinase N-lobe (Supplementary Fig. 7b).

By contrast, active Lck, lacking its regulatory domains and SH2-kinase inter-domain linker, exhibits a collapsed hydrophobic stack (Supplementary Fig. 7c). The conformational change is propagated to an inward rotation of the αC helix and formation of the salt bridge between Glu288 and Lys273 that characterises the active state of protein kinases^42^. The side chain of the invariant tryptophan (W238) is reoriented to form a hydrogen bond between the amide nitrogen of its indole ring and the main chain carbonyl of Leu303, while Lys293 in the αC helix maintains a stacking interaction with Trp238 by reorienting its side chain; together, these interactions stabilise the active conformation of the αC helix.

To investigate whether disruption of the hydrophobic stack could trigger an allosteric switch from the inactive to active conformation, we mutated the central hydrophobic amino acid in the stack contributed by the SH2-kinase linker (Itk Leu351, Src Leu255) (Figs 1a, 3a). The mutant proteins and their phosphorylated species were validated by high-resolution anion-exchange chromatography, immunoblotting, and mass spectrometry (Supplementary Fig. 1c-e, Supplementary Fig. 3c-e). Due to hyperphosphorylation of purified Itk^32K^ L351A, unphosphorylated protein was produced by *in vitro* dephosphorylation and validated by mass spectrometry (Supplementary Fig. 1f).

Mutation of Leu351 to alanine causes a reduction in compactness of Itk^32K^ as judged by a 10% increase in the radius of gyration and the maximum dimension of the particle (Fig. 3b), consistent with displacement of the inhibitory SH3-kinase interaction. Guinier analysis of the low angle portion of the scattering curve shows the clear increase in R_g_ between wild type Itk^32K^ and Itk^32K^ L351A (Supplementary Fig. 6c, Supplementary Fig. 7d). To investigate the consequences of the L351A mutation in more detail, we calculated the *ab initio* molecular envelope for Itk^32K^ L351A and compared it to the crystal structure of autoinhibited Src (ΔC-tail). We could not fit the data with the structure of Src (χ^2^ = 35.29), so we turned to rigid body modelling of the individual domains to try to better fit the scattering curve. Allowing only the SH3 domain to move did not result in an acceptable fit, but by allowing both the SH2 and SH3 domains to move freely, we were able to fit the scattering with a reasonable chi-square (Supplementary Fig. 7e). The best fit to the scattering shows a significant rotation and translation of both domains with respect to the kinase domain (Fig. 3c). Our results suggest that the L351A mutation destabilises the inhibitory interactions of both the SH3 and SH2 domains with the kinase domain, but does not lead to a wholesale displacement of the regulatory domains from the kinase domains.

To establish whether hydrophobic stack disassembly triggers a switch in nucleotide binding of the kinase domain, we measured the binding of both unphosphorylated and activation loop phosphorylated (pY511) Itk^32K^ L351A to ADP and ATP. While the affinity for ADP remained unchanged, we observed that Itk^32K^ L351A now binds ATP with comparable affinity to ADP (Fig. 3d). Activation loop phosphorylation did not significantly change the affinity for ATP, but slightly lowered the affinity for ADP (Supplementary Fig. 7f), perhaps by relieving the coordination of the beta phosphate by Arg388 in the C-lobe.

To test whether Src exhibits the same switch in nucleotide binding affinity as Itk, we tested the equivalent mutation in Src lacking its C-terminal tail (Src^32K^ΔC L255A). Due to limiting protein concentrations, we were unable to obtain a binding curve by fluorescence anisotropy, so we instead used thermal stability measurements to investigate its nucleotide-binding properties. Determination of accurate binding constants from thermal stability measurements requires knowledge of the enthalpy of unfolding. However, for a given protein, the enthalpy of unfolding is independent of the ligand being tested, so comparison of the concentrations of nucleotide required for half maximal stabilisation is valid^43^. In contrast to Src^32K^, Src^32K^ΔC L255A binds ATP approximately three times better than ADP and is stabilised to a greater extent by ATP than ADP (Fig. 3e). The affinities for ADP and ATP that we obtain by fluorescence anisotropy (Itk) or by thermal stability measurements (Src) are comparable to those obtained for the isolated Src kinase domain by isothermal titration calorimetry^44^. Taken together, our results demonstrate that disruption of the hydrophobic stack in Itk and Src triggers a conformational switch in the kinase domain that favours ATP binding.

### Nucleotide exchange permits trans-autophosphorylation of Itk and Src

**Figure 4:**
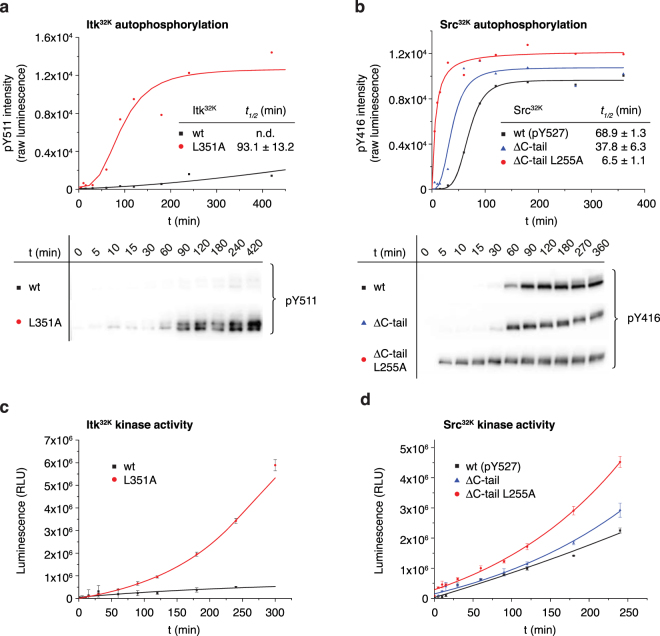
Nucleotide exchange permits trans-autophosphorylation. (**a**) Itk^32K^ autophosphorylation. Itk^32K^ and Itk^32K^ L351A were mixed with Mg^2+^ and ATP and allowed to autophosphorylate over time. Autophosphorylation was monitored with a specific antibody against the phosphorylated activation loop (pTyr511) and the results quantitated from the chemiluminescent signal. Itk^32K^ autophosphorylates on Tyr511 very slowly compared to Itk^32K^ L351A, which has a half time for maximal autophosphorylation of ~93 min. Under the same conditions, Itk^32K^ did not achieve maximal autophosphorylation. Full-length blots can be found in Supplementary Fig. 8a-b. (**b**) Src^32K^ autophosphorylation. Src^32K^, Src^32K^ΔC, and Src^32K^ L255A were mixed with Mg^2+^ and ATP and allowed to autophosphorylate over time. Autophosphorylation was monitored with a specific antibody against the phosphorylated activation loop (pTyr416) and the results quantitated from the chemiluminescent signal. Src^32K^ autophosphorylates on Tyr416 with a half time for maximal autophosphorylation of ~70 min. Under the same conditions, Src^32K^ΔC, which lacks its regulatory C-terminal tail phosphorylated itself on Tyr416 approximately 1.8-fold faster. Src^32K^ΔC L255A, lacking its C-terminal tail and mutated in its hydrophobic stack, phosphorylates almost 6 times faster than Src^32K^ΔC and more than 10 times faster than Src^32K^. Full-length blots can be found in Supplementary Fig. 8c-e. (**c**) Kinase activity assay of Itk^32K^ against an exogenous Src substrate (Cdk1 aa 6-20). Itk^32K^ is a very poor kinase against the peptide substrate, whereas Itk^32K^ L351A readily phosphorylates it. (**d**) Kinase activity assay of Src^32K^ against an exogenous Src substrate (Cdk1 aa 6-20). Removal of the C-terminal tail (Src^32K^ΔC) results in a modest activation of Src^32K^ compared with the dramatic activation seen with the hydrophobic stack mutant (Src^32K^ΔC L255A).

Having observed that hydrophobic stack disassembly promotes ATP-binding in both Itk and Src, we determined whether this also promotes autophosphorylation and activation against an exogenous substrate. Whilst wild type Itk^32K^ is a very poor kinase against Tyr511, Itk^32K^ L351A autophosphorylates very efficiently (Fig. 4a). To demonstrate that Src exhibits the same behaviour, we evaluated Tyr416 autophosphorylation of Src^32K^, Src^32K^ΔC, and Src^32K^ΔC L255A *in vitro*. Src^32K^ΔC, lacking its C-tail, exhibits only modestly higher autophosphorylation (1.8-fold). However, disruption of the hydrophobic stack in Src results in an 11-fold activation of the kinase and very efficient autophosphorylation (Fig. 4b). Notably, the slow initial rate of autophosphorylation is dramatically accelerated in both Itk^32K^ L351A and Src^32K^ΔC L255A, consistent with a lowering of the activation energy barrier to the conformational change between inactive and active states. While Itk^32K^ is a very poor kinase against a peptide derived from Cdk1, Itk^32K^ L351A readily phosphorylates it (Fig. 4c). The kinetics can be fitted with an exponential function in which the lag phase describes the activation of the kinase by autophosphorylation. Similarly, Src^32K^ is a relatively inefficient kinase, and while removal of its C-tail is modestly activating, mutation of the hydrophobic stack converts it into a very efficient enzyme (Fig. 4d).

## Discussion

Previous crystal structures of Src and Hck in complex with AMPPNP both resolved the adenosine, alpha and beta phosphates, and a single magnesium ion in the electron density, but observed poorly defined density and an uncoordinated, solvent accessible conformation for the terminal gamma phosphate, respectively. In solution, however, both Itk and Src bind to ADP with approximately an order of magnitude greater affinity. Whilst the crystal structure of Src in complex with ADP shows minimal differences to AMPPNP-bound Src, molecular dynamics calculations of the free energy of binding reinforce the strong preference for ADP.

Cellular concentrations of ADP have been measured at between 100 μM and 1.3 mM in both bacterial^45^ and mammalian cells^46,47^, while a carefully controlled mass spectrometry study found that the ratio of ATP to ADP in rat kidney tissue varied between 1.69 and 2.65^34^. In *E. coli*, the ratio of ATP to ADP in glucose-fed, exponentially growing cells is approximately 17:1^45^, which likely places an upper limit on the ratio. Given that a 9–23 fold molar excess of ATP is required to displace 50% of Mant-ADP from Src/Itk *in vitro* and independent measures of the cellular ATP/ADP ratio vary between 1.7 and 17^33,45–47^, the majority of inactive Src or Itk is likely to be ADP-bound in the cell under all metabolic conditions.

Interestingly, Src lacking its C-terminal tail retains its binding preference for ADP, while solution X-ray scattering measurements indicate that Src^ΔC^ and an SH2 domain mutant of Src both adopt the same conformation as Src and Itk, suggesting that the role of the C-tail in Src is not to lock the closed conformation. A previous SAXS and molecular dynamics study of Hck found that the solution scattering of dephosphorylated Hck could be modelled by more than 85% of Hck molecules in the autoinhibited conformation^48^. A separate molecular dynamics study of Src found that dephosphorylation of the C-terminal tail promoted higher flexibility in the SH2 and SH3 domains^49^ but, significantly, did not reveal any domain displacements. Rather, this study suggests that the energy barrier to Src activation is lowered by tail dephosphorylation, consistent with the very modest effect of tail deletion on *in vitro* activity. These observations are also consistent with an enzymological study which found that tail phosphorylation of Src does not affect the *K*_*m*_ for either ATP or substrate peptide^50^ and a study on Lck that found that tail phosphorylation did not contribute to inhibition of activation loop-phosphorylated Lck^51^. Although a structural study found that Src adopts an open conformation in the absence of tail phosphorylation^52^, this conclusion was based on crystallisation of Src in the presence of the inhibitor imatinib, which promotes a conformation of the kinase domain resembling that of active Lck (r.m.s.d. of 1.2 Å over all atoms)^38^.

In the Abl kinases, which lack a C-terminal tail, docking of the myristoylated N-terminus to a hydrophobic pocket on the C-lobe of the kinase domain was proposed to stabilise the autoinhibited conformation^53^, though a more recent SAXS study showed that a point mutant in the pocket that prevents myristoyl binding does not lead to disassembly of the regulatory domains^54^. Furthermore, recent investigations of ancestral Src kinase in unicellular choanoflagellates and filastereans have revealed that, while the C-terminal tail is present, kinase activity is not suppressed by Csk^55,56^, suggesting that phosphorylation of the C-terminal tail was a later evolutionary adaptation.

All of these observations are contrasted by cellular studies demonstrating that deletion of the tail or mutation of its phosphorylation site leads to hyperactivation of Src and cellular transformation^57–61^. Whilst the C-terminal tail of Src is of undoubted importance in the cell, we find that it has no effect on Src conformation and only a very modest effect on Src activity *in vitro*. Indeed, one can dispense with either the C-tail or the capacity to bind it, and Src still adopts the autoinhibited conformation. Therefore, although v-Src is transforming in cells, it is unlikely due to the intrinsic disassembly of its autoinhibited conformation. Indeed, the SH2 domain mutant of c-Src incapable of engaging its C-tail is not transforming^62,63^. Likewise, the Tec kinases Btk and Itk require a functional SH2 domain for their activation, despite lacking a C-tail^16,64^. Notably, while constitutively membrane localised Btk has transforming activity, mutation of its SH2 domain abrogates this activity^64^.

A distinguishing feature of the Src kinases is the presence of a membrane anchor, in contrast to the Tec kinases, which are transiently recruited upon PIP_3_ production. Consequently, Src kinases are, on average, exposed to a higher density of SH2 and SH3 domain ligands. *In vitro*, the phosphorylated C-terminal tail of Src inhibits engagement of the SH2 domain by exogenous ligands supplied in trans by a factor of ~200^50^. We therefore favour a mechanism in which the phosphorylated C-terminal tail acts as a competitive inhibitor of SH2 domain engagement with activating ligands in the cell, rather than a physical latch that maintains the closed conformation.

Another widely used proxy for Src activation is activation loop phosphorylation. We show, however, that activation loop phosphorylation of Src alone does not change its overall conformation or binding preference for ADP, indicating that the regulatory and kinase domains are still assembled in the autoinhibited conformation. Whilst the electrostatic network stabilising the inactive conformation of the activation loop is undoubtedly important^65^, its disruption does not appear to lead to a conformational switch in either the regulatory domains or the nucleotide binding site of the kinase domain, an observation consistent with a recent molecular dynamics study^66^.

The invariant tryptophan in the N-lobe of the kinase domain has previously been the focus of efforts to understand the allosteric activation of these kinases. While mutation of Trp260 in Src and Hck results in their hyperactivation^40,67^, mutation of the equivalent residue in Btk (Trp395) and Itk (Trp356) renders them inactive^68–70^. Whilst these apparently opposite observations have been used to argue that these kinases are mechanistically distinct, Trp395 of Btk is observed in the same, active conformation as Trp260 of Src and Trp238 of Lck, supporting the notion that they employ similar mechanisms to activate their respective kinase domains^71^. Recent molecular dynamics studies have also demonstrated the importance of the invariant tryptophan in stabilising the active conformation of both Src and Tec kinases^69,72^. The invariant tryptophan therefore fulfils a dual role in stabilising both the inactive and active conformations.

In order to avoid compromising the catalytic machinery in Src and Itk, we mutated a hydrophobic residue in the inter-domain linker that acts as a ‘molecular glue’ between the kinase and regulatory domains. Critically, this residue inserts its side chain into a hydrophobic stack on the back of the N-lobe of the kinase domain, which stabilises the inactive conformation of the kinase^24,40,73^. Mutation of this residue has previously been shown to abrogate Csk mediated inhibition of Src in a yeast cell-based assay^41^. We observed a dramatic switch in affinity for ATP, which is accompanied by efficient activation loop autophosphorylation and attainment of full catalytic activity. That Src and Itk bind ATP in their active conformations is not surprising, but the selective binding of ADP by the inactive kinases has not been previously observed. We speculate that this may serve to prevent promiscuous kinase activity and restrict kinase activation exclusively to situations in which the regulatory domains, and specifically the SH3 domain, have been engaged by intracellular ligands. As such, one could regard the hydrophobic stack as the ultimate regulatory node that controls the activation of Src and Tec family kinases, irrespective of activation loop phosphorylation or C-terminal tail dephosphorylation.

Whilst mutation of the linker led to a dramatic increase in binding affinity for ATP, it resulted in a very modest conformational change in Itk, consistent with regulatory domain displacement, but not complete disassembly of the regulatory and kinase domains. A putative conformation of the mutant Itk shows the SH2 domain to be in close proximity to the N-lobe of the kinase domain, though we cannot be certain of the respective domain arrangement given the resolution limits of the SAXS data. Intriguingly, the SH2 domain of c-Abl was found to enhance kinase activity through an interaction with the kinase N-lobe that stabilises the active conformation of the αC-helix, a mechanism that was also demonstrated for Fes^74^. Whilst these structures are reminiscent of active Csk^75^, the SH2 domain actually adopts distinct positions and makes different contacts with the kinase N-lobe in each structure. It is therefore unclear how and to what extent the SH2 domain may modulate catalytic activity through the N-lobe in the various family members.

**Figure 5:**
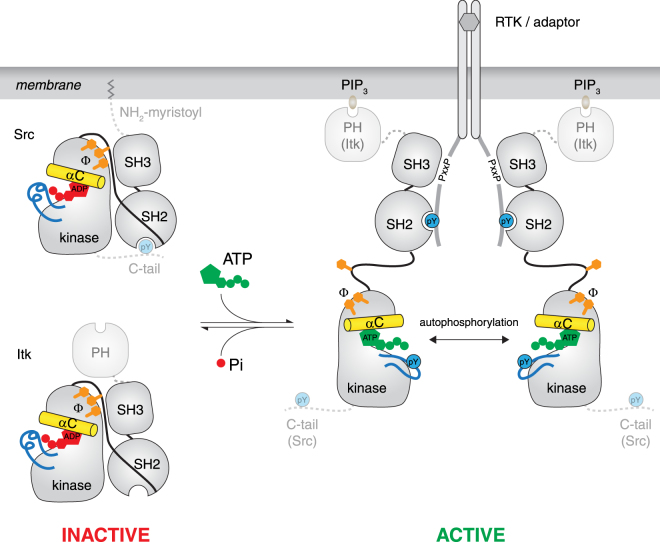
Model for the allosteric activation of Src and Tec family kinases. Src and Tec kinases adopt a closed, autoinhibited conformation in the cytosol of unstimulated cells stabilized by ADP. Activation of receptor tyrosine kinases in the membrane leads to the phosphorylation of motifs in their cytoplasmic domains and the consequent recruitment of phosphotyrosine-binding SH2-domain containing proteins, including Src and Tec kinases. Engagement of SH2 and SH3 binding motifs in the receptor or adaptor proteins leads to activation of these kinases. Displacement of the SH3 domain from contacts with the N-terminal lobe of the kinase domain disrupts a hydrophobic stack that stabilizes the inactive state, leading to a conformational change that drives the exchange of ADP for ATP, trans-autoactivation, and downstream substrate phosphorylation. Additional regulatory adaptations restrict Itk activity to PIP_3_-containing membranes on account of its regulatory PH domain, while the C-terminal tail of Src prevents spurious activation at the membrane. (yellow, αC helix; orange, hydrophobic stack; blue, activation loop; red, ADP; green, ATP; Φ, hydrophobic amino acid).

Our data are consistent with the following model (Fig. 5): In the cytosol, inactive Src and Tec kinases exist bound to ADP. Activation is driven by regulatory domain displacement by specific interactions with SH2 and SH3 domain ligands. Whilst it is clear from our data that SH3 domain displacement is a prerequisite for Src and Itk activation, it may not necessarily be the case that wholesale displacement of both SH2 and SH3 domains is required in all circumstances. SH3 domain displacement is accompanied by collapse of the hydrophobic stack on the distal surface of the N-lobe of the kinase domain and an inward rotation of the αC helix to its active conformation; repositioning of the αC helix breaks the salt bridge between Glu310 in the helix and Arg409 in the activation loop, expelling the activation loop from the catalytic cleft in the process. A salt bridge between Glu310 in the αC helix and Lys295 in the N-lobe stabilises the active conformation and creates the binding site for ATP and a second magnesium ion. The αC helix is further stabilised by the formation of a hydrogen bond between the indole ring of Trp260 and the main chain carbonyl of Leu325. In the active conformation, Src binds ATP with equal affinity to ADP, but ADP is rapidly exchanged for ATP due to the high ratio of ATP to ADP in the cell. Once loaded with ATP, Src is primed for catalytic activity, but must first immobilise its activation loop to create a surface for substrate docking. Autophosphorylation proceeds rapidly, driving immobilisation of the phosphorylated activation loop on the C-lobe of the kinase. Since ADP release is rapid and non-limiting for phospho-transfer^76^ iterative cycles of substrate phospho-transfer will be ensured as long as the regulatory domains remain engaged with their cognate ligands. While the core catalytic machinery is conserved between Itk and Src, additional regulatory features restrict their activities. In the case of Itk, the N-terminal PH domain restricts activity to PIP_3_-containing membranes. In the case of Src, which has an N-terminal membrane anchor, we propose that its C-terminal tail prevents spurious activation in the context of a high density of potentially activating ligands at the membrane.

In summary, we propose a general mechanism for the allosteric regulation of the Src and Tec kinases. We describe the basic unit of kinase control and show that the inactive state is bound and stabilised by ADP. Based on the strong sequence and structural homologies between the Src, Abl, Tec, Csk, and Fer kinases, we predict that the mechanisms of autoinhibition and kinase activation are also conserved. We propose that nature has evolved various sub-family specific adaptations, such as the C-terminal tail of Src or PH domain of Itk, that further refine the control and localisation of enzymatic activity.

## Methods

### Antibodies

The following antibodies were used in this study: Src (Cell Signaling Technology, 32G6); Src pY416 (Cell Signaling Technology, D49G4); Itk (Cell Signaling Technology, 2F12); Btk pY551 (Itk pY511) (BD Pharmingen clone 24a).

### Protein expression and purification

Human Itk^171–620^ (Itk^32K^), mouse Src^85-535^ (Src^32K^), and mouse Src^85-523^ (Src^32K^ΔC) as well as their respective point mutants were expressed as GST fusions in baculovirus-infected Sf9 insect cells. For the generation of Tyr527-phosphorylated Src^85-535^, the protein was co-expressed with human Csk using pFastBac Dual (Invitrogen). Proteins were purified by sequential glutathione-sepharose affinity chromatography, TEV cleavage, high-resolution anion exchange, and size exclusion chromatography in a final buffer of 50 mM Tris, pH 7.5, 200 mM NaCl, 1 mM TCEP, 1% (v/v) glycerol.

### Mass spectrometry

500 ng protein was dissolved in 20 mM Tris 7.6, 50 mM KCl, 1 mM TCEP and loaded on an Eksigent MicroLC column (ChromXP C4, 3 μm particle size, 300 Å pore diameter, dimensions 50 × 0.5 mm) using a Dionex Ultimate 3000 HPLC system (Thermo Scientific). The proteins were separated on a gradient from 5% to 90% acetonitrile in water and 0.1% formic acid for 30 min at a flow rate of 30 µL/min. Mass spectra were recorded on a Sciex TripleTOF 5600 instrument equipped with a Turbo V DuoSpray ion source in positive mode. Data was analysed in PeakView version 2.1 (Sciex) using the Bio Tool Kit to reconstruct the uncharged average protein mass. All other samples were diluted in 0.1% formic acid (FA) to a concentration of 10 ng/μL and 50 ng were loaded on an Aeris Widepore C4 column, 3.6 µm particle size, dimensions 2.1 × 150 mm (Phenomenex), using a Dionex Ultimate 3000 HPLC system (Thermo Scientific) with a working temperature of 55 °C, 0.1% FA as solvent A, 90% acetonitrile, 0.08% FA as solvent B. Proteins were separated in a 6 min gradient from 10 to 70% solvent B at a flow rate of 300 μL/min. Mass spectra were recorded on a Waters Synapt G2-Si equipped with a ZSpray ESI source. Data were analysed in MassLynx V 4.1 using the MaxEnt 1 process to reconstruct the uncharged average protein mass.

### Differential scanning fluorimetry

Thermal stabilities as a function of nucleotide concentration were measured by differential scanning fluorimetry (DSF). Samples contained 0.1 mg/ml protein in 50 mM Tris pH 7.5, 150 mM NaCl, 1 mM TCEP, and 5 mM MgCl_2_ with ADP, ATP, or AMPPNP concentrations ranging from 0–16 mM. Samples were measured in triplicate using a BioRad iQ™5 Multicolor Real-Time PCR Detection System. For detection of phosphorylated protein, samples were diluted and 1 ng and 15 ng were loaded to SDS-PAGE for Src and Itk proteins, respectively. Phosphorylation was measured by immunoblot.

### Fluorescence anisotropy

The binding constants for ADP and AMPPNP were determined for Src and Itk proteins by fluorescence anisotropy, using 2′/3′-Mant-labelled nucleotides (Jena Bioscience). Increasing concentrations of Itk^32K^, Itk^L351A^ or Src^32K^ were incubated with 40 μM Mant-nucleotide in 30 mM Tris pH 7.5, 200 mM NaCl, 5 mM MgCl_2_, and 1 mM TCEP. Measurements were made with a Perkin Elmer LS50B fluorimeter (λ_ex_ = 355 nm, λ_em_ = 447 nm) at 20 °C. For each data point 50–60 measurements, each with an integration time of 1 s, were averaged. For competition nucleotide displacement assays, 40–80 μM of protein was incubated with 40 μM Mant-ADP until equilibrium was established. Unlabelled nucleotide was titrated into the reaction and fluorescence anisotropy monitored. The equilibrium inhibition constant for the unlabelled nucleotide was calculated from the fitted curve.

### Small-angle X-ray scattering (SAXS)

SAXS data were collected on 0.5–4 mg/ml samples of Itk or Src in 50 mM Tris, pH 7.6, 200 mM KCl, 1 mM TCEP, 1% (v/v) glycerol, 2 mM ADP, 2 mM MgCl_2_ on BioSAXS beamline BM29, ESRF. Data reduction and analysis was performed using the BsxCuBE data collection software and the ATSAS package^77^. Scattering data for Itk^32K^ L351A and Src^32K^ R175L were collected using an online size exclusion chromatography setup on BM29. The proteins were applied to a Superdex 200 column equilibrated in 20 mM Tris, pH 8.0, 100 mM NaCl, and 1 mM TCEP and images were acquired every second for the duration of the size exclusion run. Buffer subtraction was performed by averaging 50 frames either side of the peak. The program AutoGNOM^78^ was used to generate the pair distribution function (*P(r)*) for each isoform and to determine *D*_max_ and *R*_g_ from the scattering curves (I(q) vs. q) in an automatic, unbiased manner. *Ab initio* molecular envelopes were computed by 20 iterative cycles of simulated annealing starting with a dummy atom model in DAMMIF^79^. The models were aligned, averaged, and filtered using DAMAVER^80^. The final models for Itk and Src were superimposed using SUPCOMB^81^. The theoretical scattering curve for Src was calculated from the PDB entry 2SRC using CRYSOL^82^. The agreement between the experimental scattering curves for Src^32K^, Itk^32K^, Src^32K^ΔC and Src^32K^ R175L was evaluated in terms of the chi-squared value using DATCMP. For Itk^32K^ L351A, rigid body modelling was performed using CORAL^77^ with 2SRC.pdb Δ521–533 (C-tail) as the starting model. The rigid domains of Src were defined as follows: SH3 (84-139), SH2 (148–246), kinase (260-520). The linker residues were implemented in CORAL as dummy residues. Iterative runs of CORAL were performed in which either the SH3 domain alone or both the SH3 and SH2 domains were allowed to move, while the kinase domain was fixed.

### Crystallization, data collection, and refinement

Src^32K^ at 2.6 mg/ml was co-crystallized with 2 mM ADP and 5 mM MgCl_2_ in 50 mM PIPES, pH 6.5, 1.2 M sodium tartrate, and 20 mM DTT. Crystals were cryoprotected in reservoir solution plus 22–24% glycerol and plunge frozen in liquid nitrogen. Data were collected at 100 K on ID30A3, ESRF, Grenoble. Crystals grew in space group P2_1_2_1_2_1_ as previously reported for the human apo-protein^21^, but with altered unit cell parameters in b and c (a = 51.01 Å, b = 82.97 Å, c = 105.05 Å; α = β = γ = 90°). The structure was solved by molecular replacement using 2SRC as a search model. Residues 205–212 in a loop on the surface of the SH2 domain and residues 110–117 of the SH3 domain were slightly shifted from their position in 2SRC due to lattice contacts and were therefore rebuilt. The structure was refined by iterative cycles of simulated annealing all-atom refinement in refmac. ADP and a single magnesium ion were built into clear electron density in the nucleotide-binding site and the structure refined to a final *R*_*free*_ = 26.8, *R* = 20.8 at 2.42 Å resolution.

### Molecular dynamics simulations

Molecular dynamics simulations were performed using the GROMOS simulation package^35^, using the GROMOS 54A7 parameter set to describe molecular interactions^36^. Relative binding free energies between ADP and ATP were computed using extended thermodynamic integration^37^ from simulations of the ligands free in solution or bound to the protein. For a more detailed description of the simulation setup, the reader is referred to the supplementary material.

### Autophosphorylation assay

Autophosphorylation reactions were performed in a total volume of 100 μl containing 67 nM or 1 μM Src^32K^ or Itk^32K^ respectively in 50 mM Tris pH 7.5, 100 mM NaCl, 5 mM MgCl_2_, 1 mM TCEP, and 1 mM ATP and incubated at 30 °C. 5 μl samples were taken at each time point and loaded to SDS-PAGE. Phosphorylation was measured by immunoblot. Data were fitted in Origin using a sigmoidal function to model the slow first step (autophosphorylation by unphosphorylated kinase), the fast second step (trans-phosphorylation of unphosphorylated kinase by phosphorylated kinase), and the stationary phase in which substrate (the kinase itself) has been consumed (phosphorylated).

### Kinase assay

Kinase assays were performed using the ADP-Glo kit (Promega) according to the manufacturer’s instructions. Kinase reactions contained 500 nM purified kinase, 50 μM SRC substrate (SignalChem #S30-58), and 1 mM ATP in 20 mM Tris pH 7.5, 100 mM NaCl, 1 mM TCEP, 2 mM MgCl_2_, and 0.05 mg/ml BSA. Reactions were incubated at RT for the specified time and luminescence was measured using a TECAN Infinite F500 plate reader.

### Data availability

Crystallographic coordinates have been deposited in the Protein Data Bank with accession code 6F3F.

## Electronic supplementary material

Supplementary Information
