# Supplementary material for: A switch in nucleotide affinity governs activation of the Src and Tec family kinases
Source: Sci Rep. 2017 Dec 12;7:17405. doi: 10.1038/s41598-017-17703-5 (PMC5727165; doi:10.1038/s41598-017-17703-5)
Supplement: Supplementary file 1 — Supplementary Information [file 41598_2017_17703_MOESM1_ESM.pdf]

Supplementary Information

A switch in nucleotide affinity governs activation of the Src and Tec family kinases

Freia von Raußendorf[1], Anita de Ruiter[2], and Thomas A. Leonard[1,3]

[1]Department of Structural and Computational Biology, Max F. Perutz Laboratories (MFPL), Campus Vienna Biocenter 5, 1030 Vienna, Austria

[2]Institute of Molecular Modeling and Simulation, University of Natural Resources and Life Sciences (BOKU), 1190 Vienna, Austria

[3]Department of Medical Biochemistry, Medical University of Vienna, 1090 Vienna, Austria

Correspondence: thomas.leonard@meduniwien.ac.at

Supplementary Figure 1.

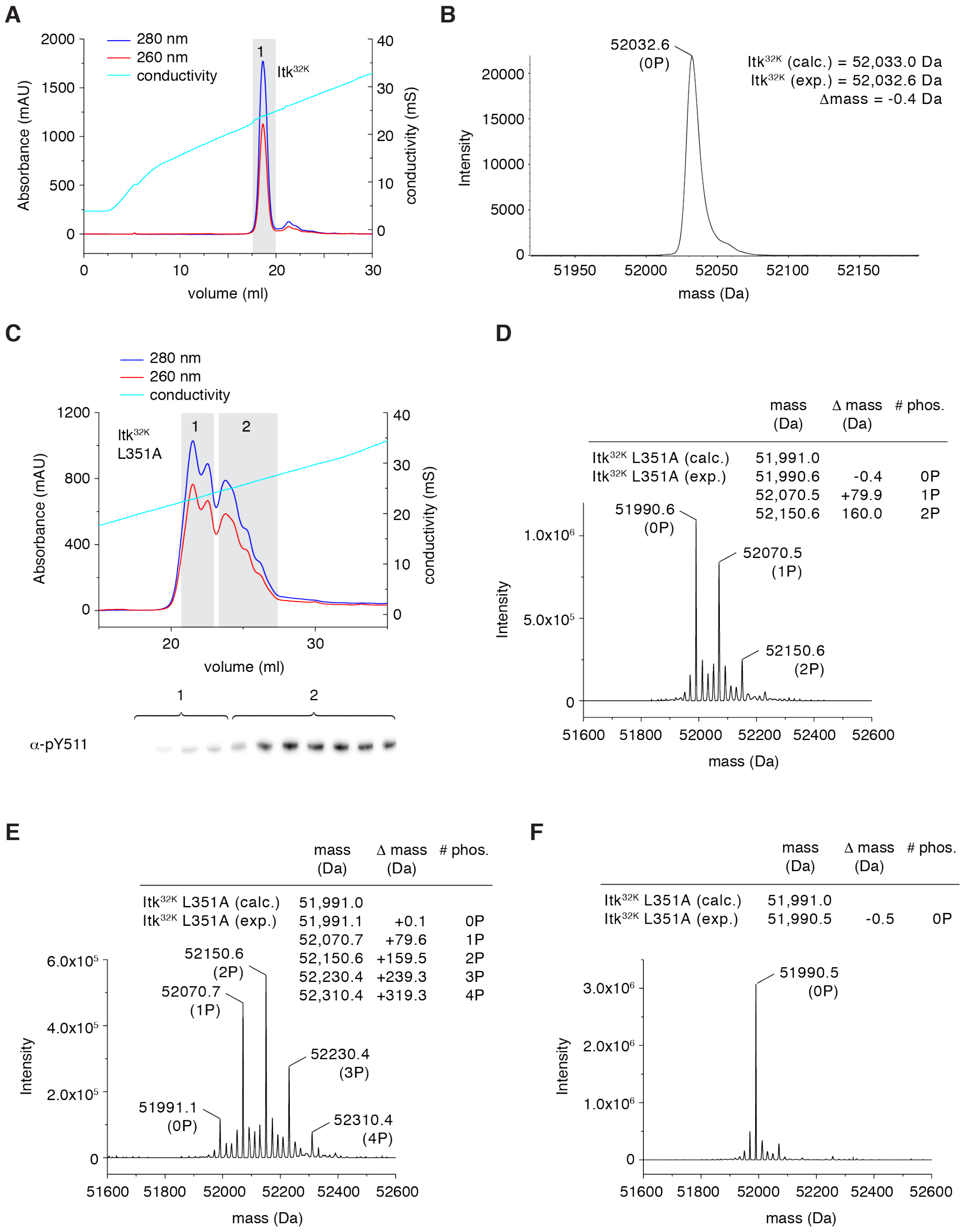

Supplementary Figure 1.

A. High-resolution anion exchange chromatography of Itk^{32K} shows a single peak.

B. Intact mass spectrometry confirmed the molecular weight of Itk^{32K} and the absence of post-translational modifications.

C. High-resolution anion exchange chromatography of Itk^{32K} L351A shows multiple phosphorylated species. Western blotting against pY511 shows the increase in activation loop phosphorylation in peaks eluting at higher salt concentration.

D. Intact mass spectrometry of 'peak' 1 confirms the presence of un-, mono-, and di-phosphorylated Itk^{32K} L351A.

E. Intact mass spectrometry of 'peak' 2 confirms the presence of un-, mono-, di-, tri, and tetrakis-phosphorylated Itk^{32K} L351A. Only a small fraction is unphoshorylated.

F. Intact mass spectrometry of in vitro dephosphorylated Itk^{32K} L351A confirms the presence of a single un-phosphorylated species.

Supplementary Figure 2.

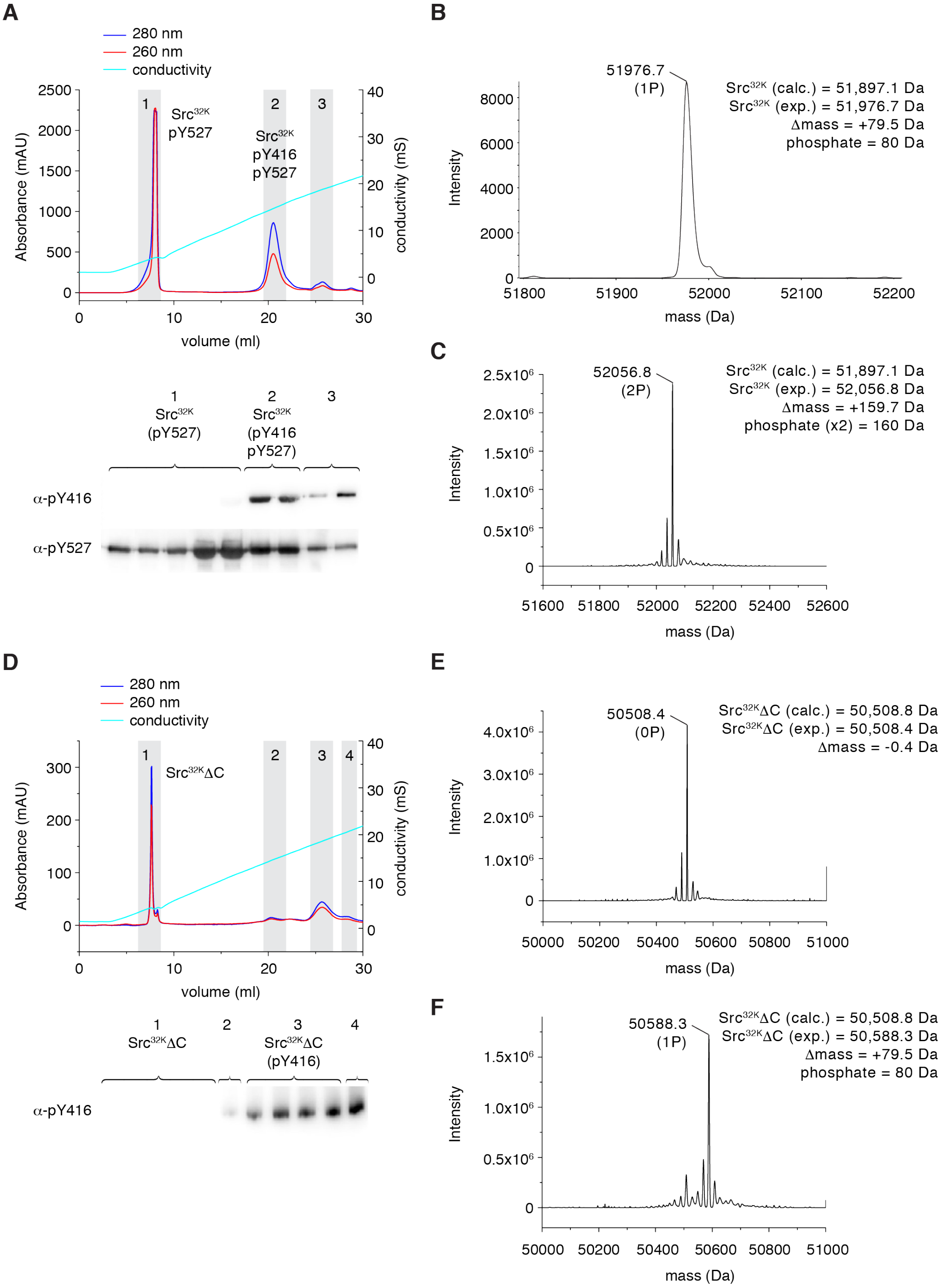

Supplementary Figure 2.

A. High-resolution anion exchange chromatography of Src^{32K} shows two major peaks. Western blotting with anti-pY527 and anti-pY416 antibodies

showed the peaks to correspond to mono- (pY527) and di- (pY416, pY527) phosphorylated Src^{32K}.

B. Intact mass spectrometry confirmed the molecular weight of peak 1 and the presence of a single phosphate.

C. Intact mass spectrometry confirmed the molecular weight of peak 2 and the presence of two phosphates.

D. High-resolution anion exchange chromatography of $Src^{32K}\Delta C$ shows one major peak (peak 1) and one small peak (peak 3). Western blotting against pY416 shows the presence of activation loop phosphorylation in peak 3 but not peak 1.

E. Intact mass spectrometry confirmed the molecular weight of $Src^{32K}\Delta C$ (peak 1).

F. Intact mass spectrometry confirmed the molecular weight of $Src^{32K}\Delta C$ (peak 3) and the presence of a single phosphate.

Supplementary Figure 3. Characterization of Src proteins (continued).

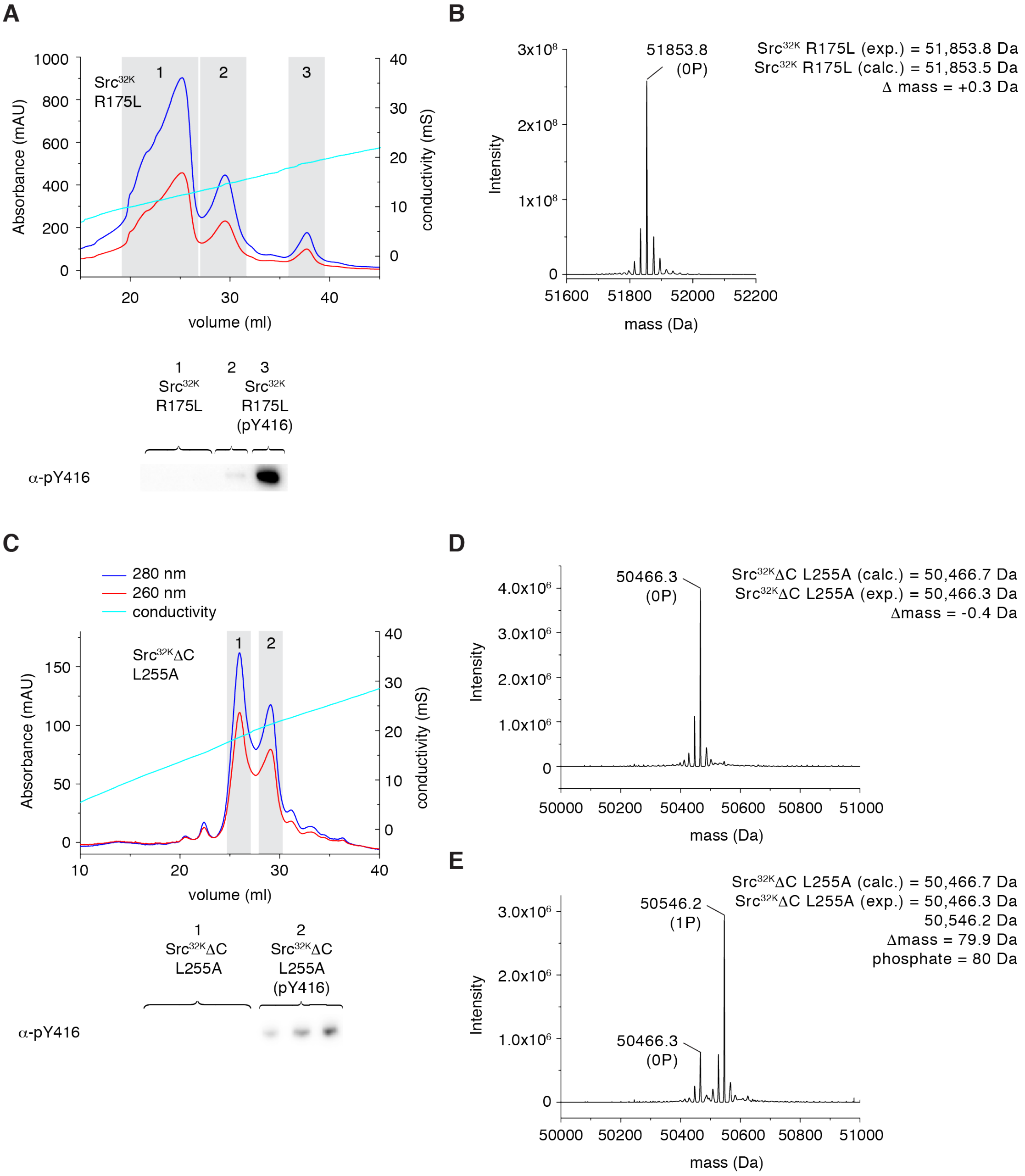

Supplementary Figure 3.

A. High-resolution anion exchange chromatography of Src^{32K} R175L. Peak 3 is phosphorylated on Y416.

B. Intact mass spectrometry of Src^{32K} R175L peak 1 confirms the presence of a single un-phosphorylated species.

C. High-resolution anion exchange chromatography of Src^{32K} L255A shows two main peaks. Western blotting against pY416 shows peak 2 to contain Y416-phosphorylated Src^{32K}.

D. Intact mass spectrometry of Src^{32K} L255A peak 1 confirms the presence of a single un-phosphorylated species.

E. Intact mass spectrometry of Src^{32K} L255A peak 2 confirms the presence of a mono-phosphorylated species and a small amount of un-phosphorylated protein.

Supplementary Figure 4. Src and Itk bind to ADP with higher affinity than ATP.

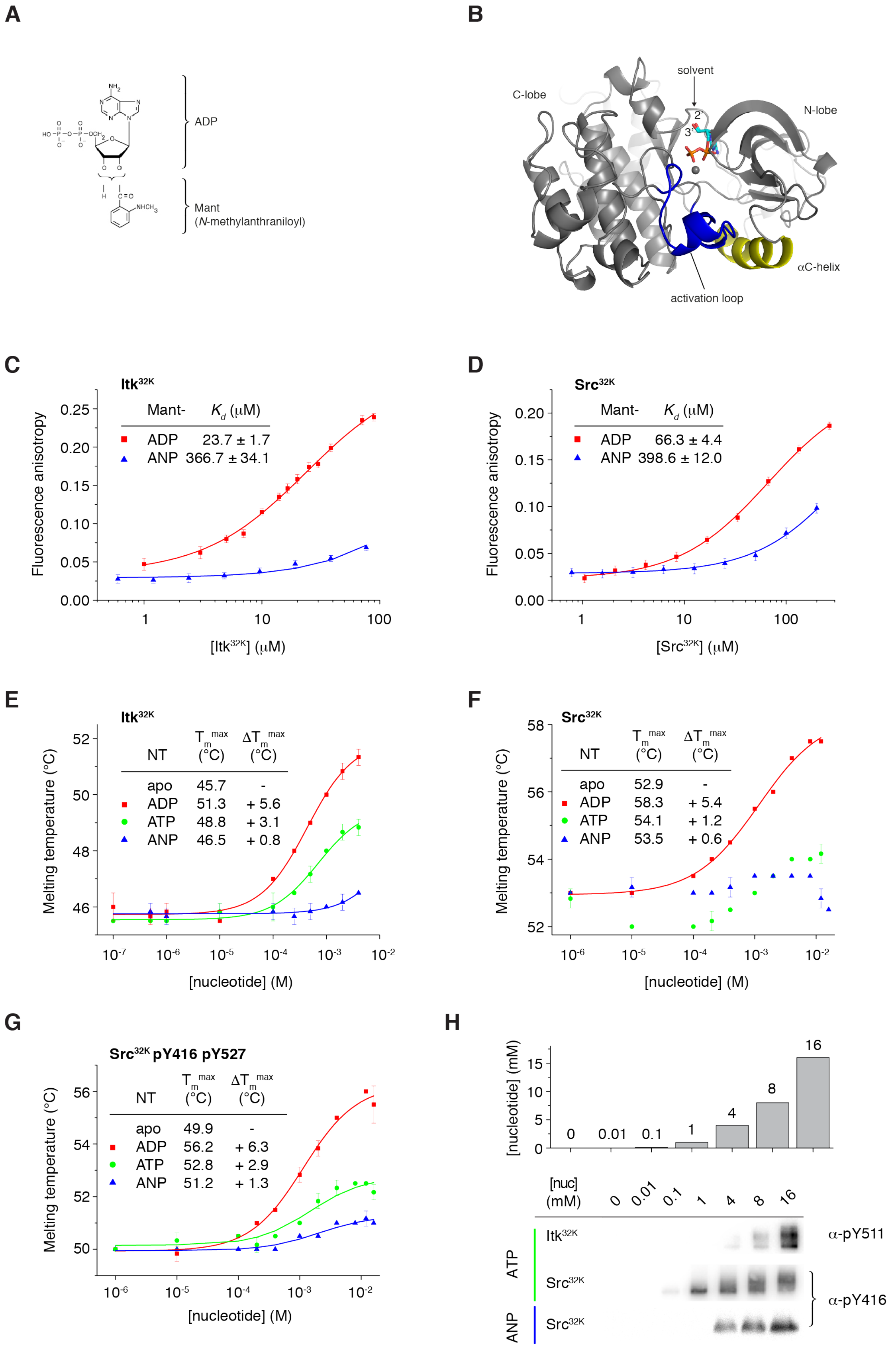

Supplementary Figure 4.

A. Chemical structure of Mant-ADP.

B. Structure of Src kinase domain bound to ADP indicates that the 2' and 3' hydroxyls of the ribose ring project into the solvent. Modification with Mant at either site does not generate any steric clashes or additional contacts.

C. Determination of the binding constants of Itk^{32K} for ADP and AMPPNP using fluorescence anisotropy of Mant-labeled nucleotides. Itk^{32K} binds to ADP with a K_d = 24 μM, with the binding to AMPPNP approximately 15-fold weaker.

D. Determination of the binding constants of Src^{32K} for ADP and AMPPNP using fluorescence anisotropy of Mant-labeled nucleotides. Src^{32K} binds to ADP with a K_d = 66 μM, with the binding to AMPPNP approximately 6-fold weaker.

E. Thermal stability measurements of Itk^{32K} in the presence of increasing concentrations of nucleotides. Itk^{32K} exhibits higher thermal stability and binds more tightly to ADP than either ATP or AMPPNP.

F. Thermal stability measurements of Src^{32K} in the presence of increasing concentrations of nucleotides. Src^{32K} exhibits higher thermal stability and binds more tightly to ADP than ATP or AMPPNP.

G. Thermal stability measurements of Src^{32K} pY416 (peak 2, Supplementary Figure 2A, C) in the presence of increasing concentrations of nucleotides. Src^{32K} pY416 exhibits higher thermal stability with ADP than ATP.

H. Src^{32K} becomes rapidly autophosphorylated during the course of thermal stability measurements in the presence of ATP. Western blots against the

phosphorylated activation loops of Itk (pY511) and Src (pY416) for samples measured at each nucleotide concentration.

Supplementary Figure 5. Crystal structure of Src in complex with magnesium-ADP.

a

Data Collection and Refinement Statistics

Data collection	
spacegroup	$P2_12_12_1$
unit cell (a, b, c) (Å)	51.01, 82.97, 105.05
wavelength (Å)	0.968
resolution (Å)	52.53 - 2.42 (2.51-2.42)
observations	70,666
unique reflections	17,601
completeness	99.5 (99.5)
multiplicity	4.0
$I/\sigma(I)$	6.1 (1.5)
R_{pim}	0.143 (0.831)
CC(1/2)	0.997 (0.258)
Structure refinement	
Resolution range	52.53 - 2.42
R-factor, R_{free} (%)	20.8, 26.8
model	81-529
r.m.s. deviations	
bond lengths (Å)	0.002
bond angles (°)	0.583

b

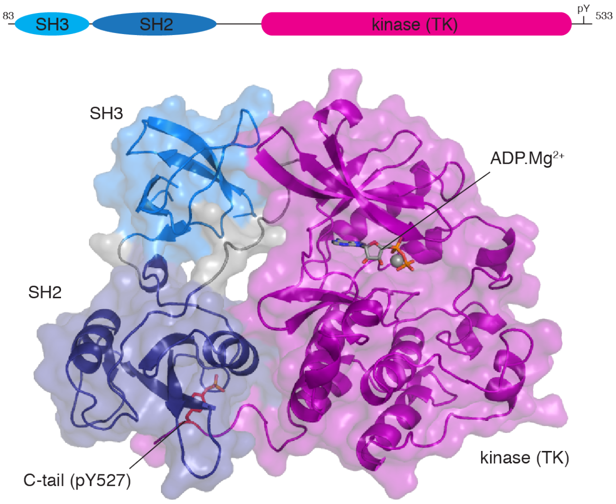

c

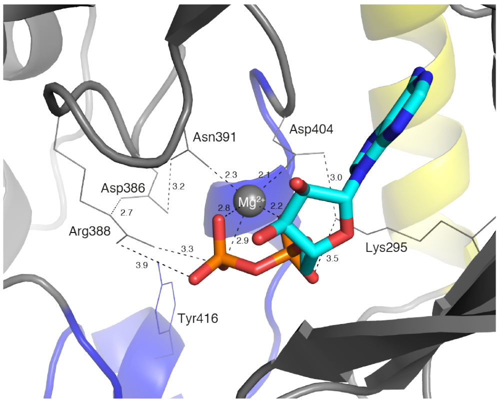

d

Electrostatic and hydrogen bond network of Src[32K]

donor		acceptor		distance (Å)
Lys295	NZ	Asp404	OD1	3.0
	NZ	ADP	O1A	3.5
Mg^{2+}	MG	Asp404	OD2	2.1
	MG	ADP	O2A	2.2
	MG	ADP	O1B	2.9
	MG	ADP	O3B	2.8
	MG	Asn391	OD1	2.3
Asn391	ND2	Asp386	OD2	3.2
Arg388	NE	Asp386	OD1	2.7
	NH2	ADP	O1B	3.3
	NH1	ADP	O2B	3.9

e

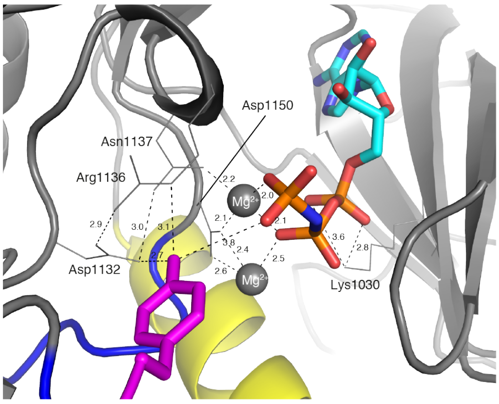

f

Electrostatic and hydrogen bond network of IRK

donor		acceptor		distance (Å)
Lys1030	NZ	ATP	O1A	2.8
	NZ	ATP	O2A	3.6
Mg^{2+}	MG1	Asp1150	OD2	2.1
	MG2	Asp1150	OD2	2.4
	MG2	Asp1150	OD1	2.6
	MG1	ADP	O2B	2.1
	MG2	ADP	O2B	2.5
	MG1	ADP	O2G	2.0
	MG1	Asn1137	OD1	2.2
Asn1137	ND2	Asp1132	OD2	3.0
Tyr10	OH	Arg1136	NE	3.1
	OH	ATP	O3G	3.8
	OH	Asp1132	OD2	2.7
Arg1136	NH2	Asp1132	OD1	2.9

Supplementary Figure 5.

A. Data collection and refinement statistics for Src in complex with ADP.Mg^{2+}.

B. Overall structure of Src^{32K} pY527 in complex with ADP.Mg^{2+}. The structure is similar to that of 2SRC, but shows clear electron density for ADP and a single magnesium ion. The phosphorylated tail and activation loop are both ordered in the structure.

C. Electrostatic and hydrogen bond network of autoinhibited Src^{32K} in the presence of ADP and a single magnesium ion.

D. Tabulated bond donors and acceptors and their distances in C.

E. Electrostatic and hydrogen bond network of insulin receptor kinase in the presence of a non-hydrolyzable ATP analog, AMPPNP, two magnesium ions, and a peptide substrate (PDB: 1IR3).

F. Tabulated bond donors and acceptors and their distances in E.

Supplementary Figure 6. Itk adopts a Src-like autoinhibited conformation.

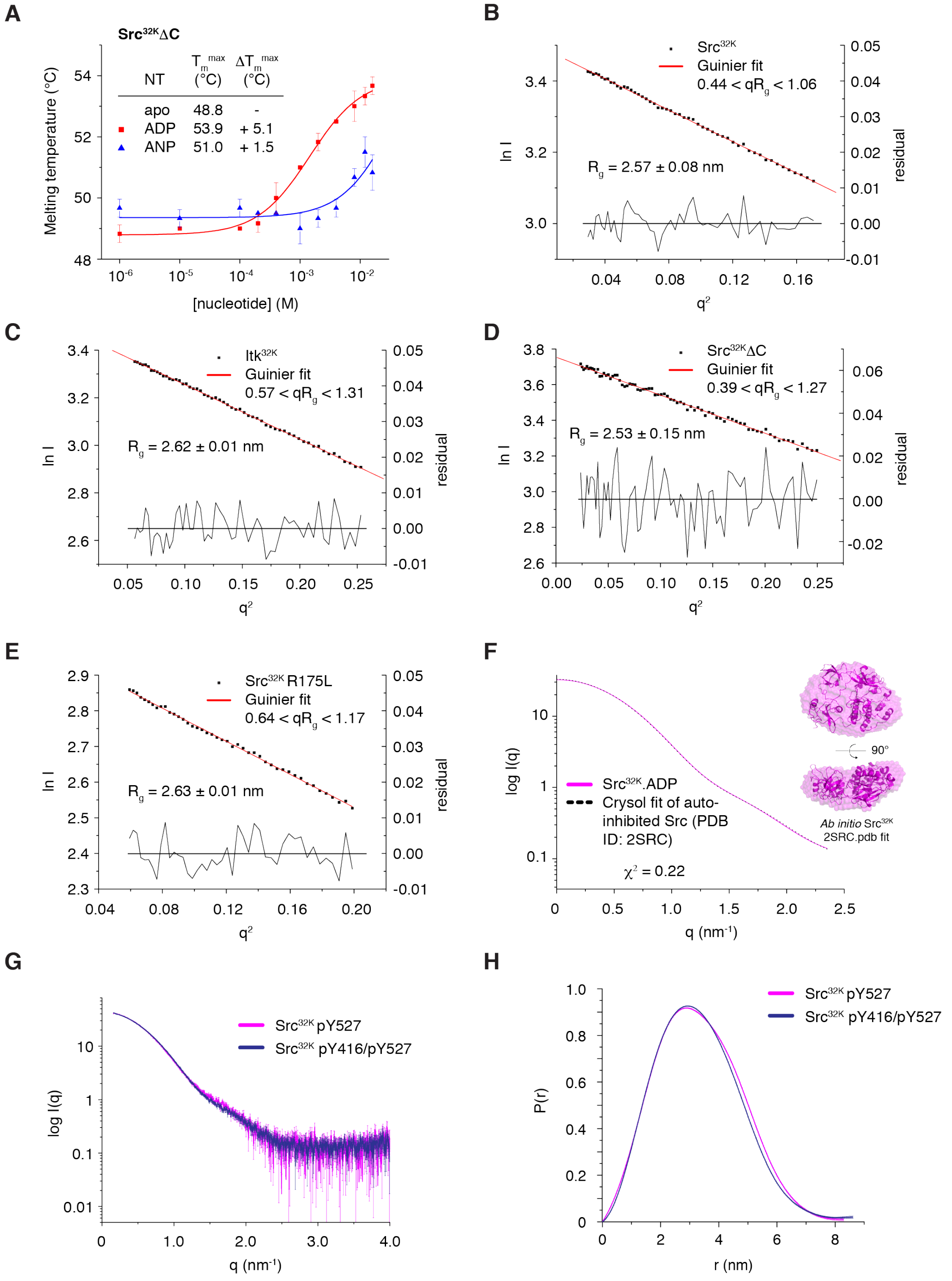

I

	Rg (Guinier) (nm)	Rg (Gnom) (nm)	D_{max} (Gnom) (nm)	χ^2 (ref. = Src32K)
Src32K pY416/pY527	2.53 ± 0.02	2.55	8.3	1.51
Src32K pY527	2.57 ± 0.06	2.56	8.2	-

Supplementary Figure 6.

A. Thermal stability measurements of $Src^{32K}\Delta C$ in the presence of increasing concentrations of nucleotides. $Src^{32K}\Delta C$ exhibits higher thermal stability and binds more tightly to ADP than AMPPNP.

B. Guinier analysis of the low-angle region of the small angle X-ray scattering curve of Src^{32K}.

C. Guinier analysis of the low-angle region of the small angle X-ray scattering curve of Itk^{32K}.

D. Guinier analysis of the low-angle region of the small angle X-ray scattering curve of $Src^{32K}\Delta C$.

E. Guinier analysis of the low-angle region of the small angle X-ray scattering curve of Src^{32K} R175L.

F. Fit of the theoretical scattering curve of Src to the DAMMIF *ab initio* calculated envelope. The χ^2 value of 0.22 indicates close to perfect agreement. Inset: *ab initio* model of Src^{32K} and docking of autoinhibited Src (2SRC.pdb) into the molecular envelope.

G. Small angle X-ray scattering curves for Src^{32K} pY527 (magenta), and Src^{32K} pY416 pY527 (purple).

H. Pair distribution functions for Src^{32K} pY527 (magenta), and Src^{32K} pY416 pY527 (purple) indicate that the particles exhibit almost identical radii of gyration and maximum dimensions.

I. Table of physical parameters obtained from the scattering curves for Src^{32K} pY527 and Src^{32K} pY416 pY527.

Supplementary Figure 7. A hydrophobic stack controls Src and Itk kinase activation.

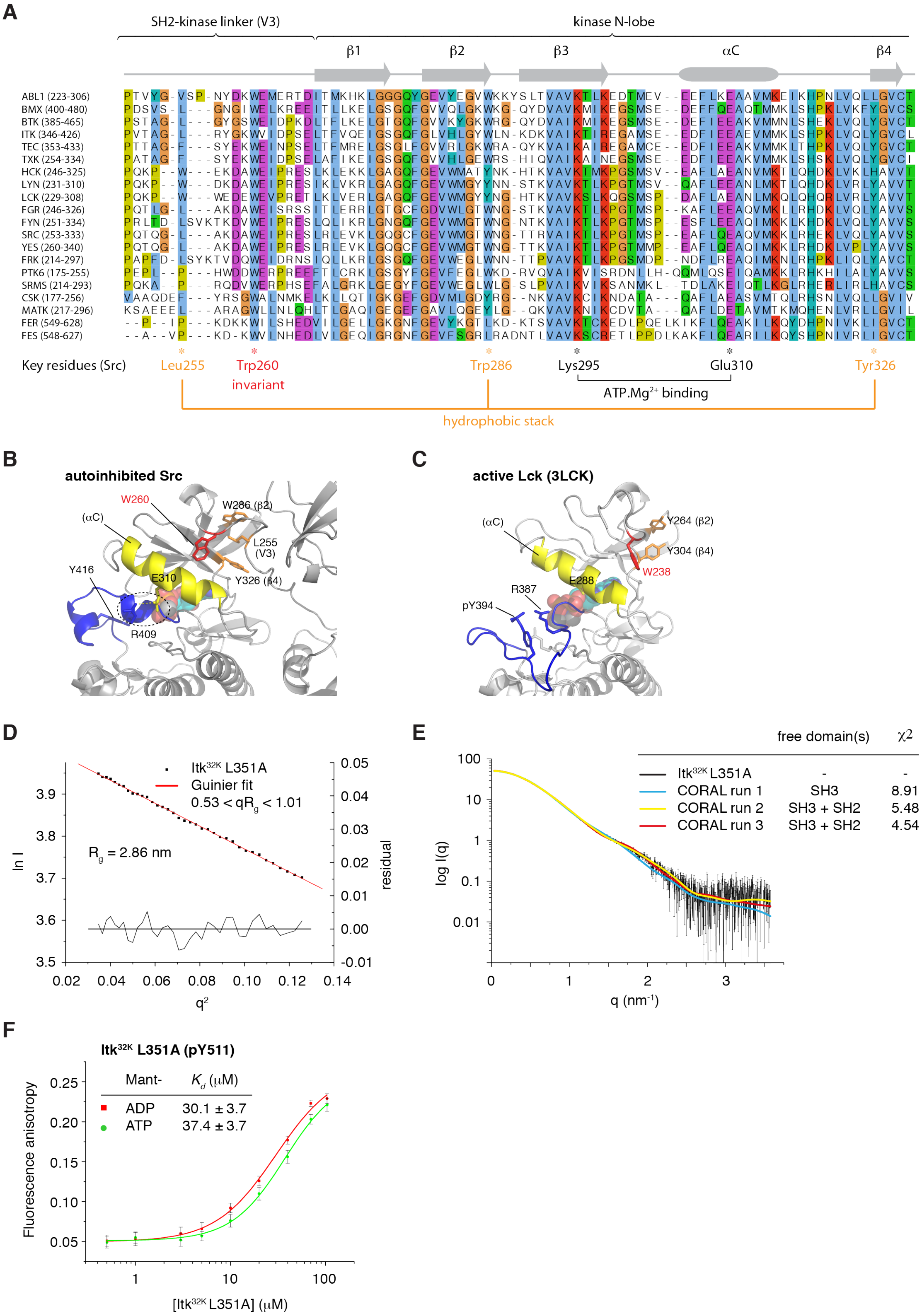

Supplementary Figure 7.

A. Sequence alignment of the SH2 linker-kinase N-lobe, illustrating the position and conservation of amino acids involved in stabilizing the inactive or active states of Src family tyrosine kinases.

B. Architecture of the hydrophobic stack in autoinhibited Src. The stack is formed on the distal surface of the N-lobe of the kinase domain by insertion of a hydrophobic residue from the SH2-kinase inter-domain linker (Leu255) between two hydrophobic side chains on the surface of the N-lobe (Trp286 and Tyr326). The intact stack maintains the αC helix in the 'out' conformation in which Trp260 inserts its side chain into a hydrophobic pocket formed between the aC helix and the β-sheet.

C. Architecture of the hydrophobic stack in active Lck. The central amino acid in the stack (Trp233) has been removed as a consequence of the absence of the regulatory SH3 and SH2 domains and the SH2-kinase inter-domain linker. The stack collapses, and the bottom residue in the stack, Tyr304, reorients to pack against the hydrophobic surface on the N-lobe exposed by removal of Trp233. The αC helix rotates to form a salt bridge between Glu288 and Lys273 in the N-lobe. Trp238 stabilizes the active conformation by forming a hydrogen bond between its indole amide and the main chain carbonyl of Leu303. Phosphorylation of the activation loop (pTyr394) results in its chelation on the C-lobe of the kinase domain by Arg363 and Arg387.

D. Guinier analysis of the low-angle region of the small angle X-ray scattering curve of Itk^{32K} L351A gives a radius of gyration of 2.86 nm.

E. Rigid body modeling of Itk^{32K} L351A. Best fits to the experimental scattering were obtained only when both the SH3 and SH2 domains were allowed to move with respect to the kinase domain (yellow and red curves).

F. Determination of the binding constants of activation loop-phosphorylated (pTyr511) Itk^{32K} L351A for nucleotides. Phosphorylated Itk^{32K} L351A binds ADP (30 μM) and ATP (37 μM) with almost identical affinities.

Supplementary Figure 8. Full-length blots of data presented in Figure 4.

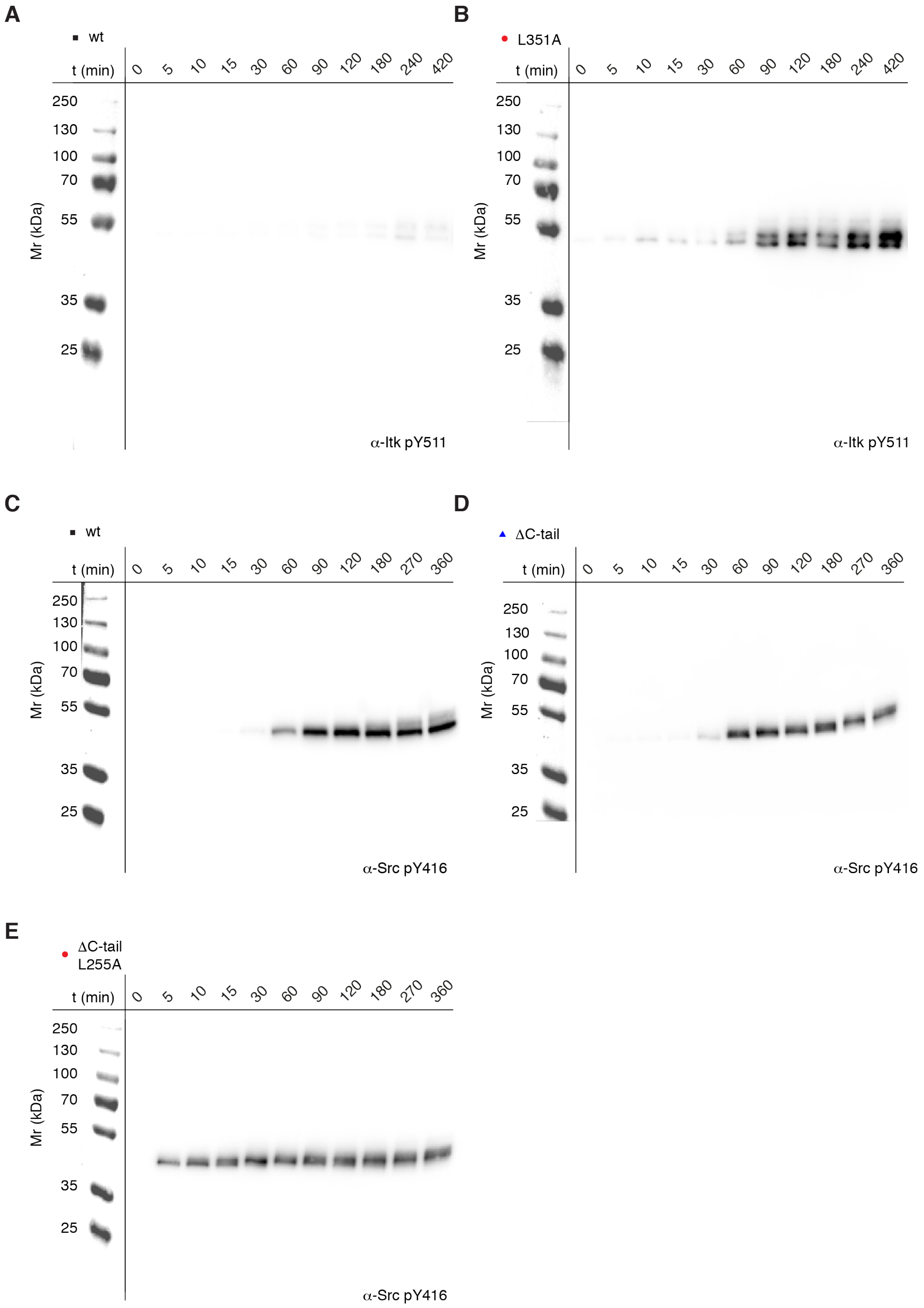

Supplementary Figure 8. Full-length blots presented in Figure 4A-B.

Supplemental Experimental Procedures

Molecular dynamics simulations

Molecular dynamics (MD) simulations in combination with free energy calculations are used to determine the relative binding affinity of ADP and ATP to Src kinase. The relative binding free energy between is defined as

$$\Delta\Delta G_{bind} = \Delta G_{bind}^{ADP} - \Delta G_{bind}^{ATP}$$

However, the actual binding processes of ADP or ATP moving from bulk water to the bound state within Src kinase is computationally rather expensive to calculate. Instead we make use of the thermodynamic cycle as shown in Figure 3E. Because free energy is a state function, we can determine the relative binding free energy also from the vertical arrows, where we convert ADP to ATP both when in solution and when bound to Src kinase:

$$\Delta\Delta G_{bind} = \Delta G_{ADP->ATP}^{free} - \Delta G_{ADP->ATP}^{bound}$$

The calculation of the free energy difference along the vertical arrows are performed with Thermodynamic Integration (TI) [1].

A slightly modified version of the GROMOS11 simulation package (version 1.3.0) [2], together with the gromos 54A7 force field [3] was used for all MD simulations. The modifications to the code include the pre-calculation of energy values and derivatives to facilitate the use of extended TI.9. Initial coordinates for the protein simulation were obtained from the crystal structure of Src kinase with bound ADP. The simulation was restricted to the stable kinase domain (residues 250-518) in order to reduce simulation time. The system was solvated in a rectangular box filled with 10,933 SPC water molecules [4] together with 3 Na^{+} ions to neutralize the ADP-Src complex. The crystal shows a Mg^{2+} ion bound to

ADP. During the simulation, this ion was kept in place by harmonic distance restraints to the coordinating groups in both the protein and ADP/ATP, using a force constant of 2000 kJ/mol/nm^2. For the simulation of ADP/ATP in water, the question is if the magnesium ion should be present as well. It could be argued that both ADP and ATP are very likely to be in complex with at least one magnesium ion in solution. The simulations in water consisted of a cubic box filled with 1,327 SPC water molecules. The magnesium ion was again kept in place with harmonic distance restraints to atoms O2A, O1B and O3B in ADP/ATP. In solution, the remaining coordination sites were occupied by water molecules.

Initial velocities were generated from a random Maxwell-Boltzmann distribution at 60 K. The system was slowly thermalized in discrete steps of 60 K and 20 ps simulations at each temperature. At the same time the force constant of initial position restraints on the solute were relaxed by a factor of 10 at each temperature, starting from 2.5×10^4 kJ mol^{-1} nm^{-2}. Upon reaching 300 K, the position restraints were turned off and rototranslational constraints were turned on. In the last 40ps of equilibration constant pressure was introduced through isotropic weak coupling [5] with a compressibility of 4.575×10^{-4} (kJ mol^{-1} nm^{-3})$^{-1}$ and a relaxation time of 0.5 ps. Temperature was kept constant using the weak coupling method [5] with a relaxation time of 0.1 ps and separate temperature baths for the solute and solvent. The SHAKE algorithm [6] was used to keep all bond lengths constant with a geometric accuracy of 1×10^{-4} (the only exception being the bond in the terminal OH group of ADP, which is changed into an O-P bond in ATP; this bond is treated as a standard (perturbed) GROMOS quartic bond). A triple range cutoff scheme is applied for the calculation of long-range interactions, for which a pairlist was generated every 5th time step. Within 0.8

nm, all interactions are calculated from the pairlist, between 0.8 and 1.4 nm, the interactions are calculated with each pairlist update and kept constant in between. A reaction field contribution [7] was used for interactions beyond 1.4 nm, with a dielectric constant of 61 as appropriate for the SPC water model [8].
TI calculations were based on a single topology approach in which the ADP phosphates were initially extended by the appropriate dummy atoms. During the TI, the dummy atoms were turned into the corresponding atoms in the gamma phosphate as a function of the coupling parameter lambda, using 11 equidistant lambda points in combination with the extendedTI protocol [9] to obtain smoother curves from fewer lambda points. This modification involves the introduction of an additional negative charge to the system. This, in principle, necessitates the use of corrections for finite size effects and methodological aspects [10]. However, the corrections for introducing a negative charge using the group-based reaction-field cutoff scheme largely cancel and are not expected to change the outcome of the calculations significantly.

The simulations in the protein were 2 ns long, whereas in water 1 ns were enough to converge the results. Statistical error estimates on the extended TI profiles were obtained by bootstrapping with 100 repeats. In the bound state, the thermodynamic integration was performed after every 0.5 ns per lambda value, leading to the following integrated values: -254.7 ± 1.6 kJ/mol after 0.5 ns/λ; -258.6 ± 1.3 kJ/mol after 1.0 ns/λ; -261.7 ± 1.2 kJ/mol after 1.5 ns/λ; 262.8 ± 1.0 kJ/mol after 2.0 ns/λ. Statistical uncertainties and fluctuations due to prolongation of the simulation length and significantly smaller than the resulting free energy of binding, indicating a sufficient convergence.

1 Kirkwood, J. G. Statistical mechanics of fluid mixtures. *J Chem Phys* **3**, 300-313, doi:Doi 10.1063/1.1749657 (1935).

2 Schmid, N., Christ, C. D., Christen, M., Eichenberger, A. P. & van Gunsteren, W. F. Architecture, implementation and parallelisation of the GROMOS software for biomolecular simulation. *Comput Phys Commun* **183**, 890-903, doi:10.1016/j.cpc.2011.12.014 (2012).

3 Schmid, N. *et al.* Definition and testing of the GROMOS force-field versions 54A7 and 54B7. *Eur Biophys J Biophy* **40**, 843-856, doi:10.1007/s00249-011-0700-9 (2011).

4 Berendsen, H. J. C., Postma, J. P. M., Van Gunsteren, W. F. & Hermans, J. Interaction Models for Water in Relation To Protein Hydration. *Intermolecular Forces (Reidel, Dordrecht, The Netherlands)*, 331-342 (1981).

5 Berendsen, H. J. C., Postma, J. P. M., Vangunsteren, W. F., Dinola, A. & Haak, J. R. Molecular-Dynamics with Coupling to an External Bath. *J Chem Phys* **81**, 3684-3690, doi:Doi 10.1063/1.448118 (1984).

6 Ryckaert, J. P., Ciccotti, G. & Berendsen, H. J. C. Numerical-Integration of Cartesian Equations of Motion of a System with Constraints - Molecular-Dynamics of N-Alkanes. *J Comput Phys* **23**, 327-341, doi:Doi 10.1016/0021-9991(77)90098-5 (1977).

7 Tironi, I. G., Sperb, R., Smith, P. E. & Vangunsteren, W. F. A Generalized Reaction Field Method for Molecular-Dynamics Simulations. *J Chem Phys* **102**, 5451-5459, doi:Doi 10.1063/1.469273 (1995).

8 Heinz, T. N., van Gunsteren, W. F. & Hunenberger, P. H. Comparison of four methods to compute the dielectric permittivity of liquids from molecular

dynamics simulations. *J Chem Phys* **115**, 1125-1136, doi:Doi 10.1063/1.1379764 (2001).

9 de Ruiter, A. & Oostenbrink, C. Extended Thermodynamic Integration: Efficient Prediction of Lambda Derivatives at Nonsimulated Points. *Journal of Chemical Theory and Computation* **12**, 4476-4486, doi:10.1021/acs.jctc.6b00458 (2016).

10 Reif, M. M. & Oostenbrink, C. Net charge changes in the calculation of relative ligand-binding free energies via classical atomistic molecular dynamics simulation. *J Comput Chem* **35**, 227-243, doi:10.1002/jcc.23490 (2014).
